# Response of the authors to the letter to the editor regarding “Development of a machine learning-based predictive model for long-term adverse outcomes in neonatal bacterial meningitis”

**DOI:** 10.1016/j.jped.2026.101525

**Published:** 2026-03-16

**Authors:** Ying Chen, Shengpei Wang, Jing Wu, Chi Wang, Ying Li, Peicen Zou, Ruiqi Xiao, Na Zhang, Huiguang He, Yajuan Wang

**Affiliations:** a Capital Institute of Pediatrics, Department of Neonatology, Beijing, China; b Chinese Academy of Medical Sciences & Peking Union Medical College, Beijing, China; c Chinese Academy of Sciences, Institute of Automation, Laboratory of Brain Atlas and Brain-inspired Intelligence, Beijing, China; d Chinese Academy of Sciences, Institute of Automation, State Key Laboratory of Brain Cognition and Brain-inspired Intelligence Technology, Beijing, China; e Capital Medical University, Capital Institute of Pediatrics, Capital Center for Children's Health, Center for Evidence-Based Medicine, Beijing, China; f Capital Medical University, Capital Institute of Pediatrics, Capital Center for Children's Health, Hemangioma and Interventional Vascular Center, Beijing, China; g Seventh Medical Center of PLA General Hospital, Faculty of Pediatrics, Department of Neonatology, Beijing, China

Dear Editor,

Thank you very much for your thoughtful and insightful comments on our manuscript, “Development of a machine learning–based predictive model for long-term adverse outcomes in neonatal bacterial meningitis.” [1]

We sincerely appreciate your recognition of the study’s clinical relevance and methodological strengths, and we are grateful for your constructive suggestions, which have helped us reflect deeply on the model’s limitations and future directions.

Below, we address your key points in detail:

First, we fully agree that the single-center design and modest final sample size (*N* = 139) represent limitations of our study. Neonatal bacterial meningitis (NBM) is a rare but devastating condition, and assembling a large, prospectively followed cohort within a single institution over five years is inherently challenging. Our cohort comprises consecutive cases from our center over the past five years, carefully curated to ensure clinical homogeneity and complete follow-up (≥ 1 year). All lost-to-follow-up cases were excluded to minimize outcome misclassification. While internal train–test splitting and cross-validation provide a reasonable initial assessment of model performance and support feature selection and hyperparameter tuning, we acknowledge they cannot substitute for external validation. As you rightly emphasized, transportability across different populations, referral patterns, microbial etiologies, and care settings must be evaluated before clinical deployment. We are pleased to inform you that we are already collaborating with multiple tertiary neonatal centers to collect external validation data, and we plan to report these results in a follow-up study.
Second, we wholeheartedly agree that high discrimination without adequate calibration can lead to misleading absolute risk estimates, potentially undermining clinical decision-making and family counseling. To address this, we deliberately reported a comprehensive set of performance metrics beyond AUROC, including the Brier score, calibration-in-the-large, decision curve analysis (DCA), and precision–recall AUC. In future work, we will supplement these results with calibration plots to further enhance the interpretability of the model’s calibration performance. Indeed, while logistic regression achieved a high AUROC, its relatively poor Brier score (≈0.235) indicated overconfidence in its risk predictions. In contrast, the random forest model offered comparable discrimination with substantially better calibration (Brier ≈0.123), which guided our choice of the final predictive tool. Going forward, we will explore recalibration techniques (e.g., Platt scaling or isotonic regression) during external validation if needed.
Third, we share your strong commitment to research transparency. Adherence to reporting standards such as TRIPOD is essential for scientific rigor and clinical translation. Once we have finalized the supplementary analyses and organized the code, we commit to making the following materials publicly available:

• Full model specifications (including feature weights or algorithm parameters);
• An implementable risk calculator (e.g., in the form of a web tool);
• The analysis code and preprocessing scripts, which will be deposited on GitHub.

We believe these steps will substantially improve reproducibility and facilitate independent validation.

Fourth, your observation regarding consensus-based feature selection possibly favoring more complex models is astute. Our rationale was to enhance robustness by integrating results from multiple selection methods, thereby reducing dependence on any single algorithm’s idiosyncrasies. While this may increase model complexity, we prioritized clinical interpretability—ensuring that all selected features (e.g., CSF white blood cell count, protein level, seizures, and imaging findings) are routinely available and biologically plausible.

Regarding class imbalance (32.4 % adverse outcomes), we acknowledge this as a limitation. To mitigate its impact, we optimized the decision threshold using Youden’s index to balance sensitivity and specificity, rather than defaulting to a 0.5 cutoff. We agree that more sophisticated strategies — such as cost-sensitive learning or synthetic minority oversampling — deserve exploration. These will be evaluated during our external validation phase, with sensitivity analyses focused on overall predictive utility rather than discrimination alone.

Finally, our composite endpoint — encompassing death, persistent imaging abnormalities, and neurodevelopmental sequelae—was intentionally designed to reflect clinically meaningful long-term harm. Among the 45 adverse-outcome cases (32.4 % of the cohort): (1) 5 (3.6 %) died; (2) 40 (28.8 %) survived with ≥ 1 neurological sequelae (totaling 73 complications), including motor deficits (11.5 %), global developmental delay (10.8 %), language disorders (9.4 %), and epilepsy (8.6 %); (3) 12 (8.6 %) exhibited persistent structural abnormalities on neuroimaging (e.g., hydrocephalus, encephalomalacia), with 11 of these also showing functional impairments.

Critically, all outcomes were adjudicated using objective evidence:

• Developmental delay required formal Gessell developmental scale scores;
• Seizures required clinical documentation plus EEG confirmation;
• Imaging findings were based on official radiology reports with longitudinal comparison;
• All endpoint assignments were independently reviewed by two senior pediatric neurologists.

Information was gathered via both in-person follow-up and structured telephone interviews; however, telephone reports alone were never sufficient — they triggered retrieval and verification of medical records. Furthermore, we excluded all lost-to-follow-up cases to avoid outcome misclassification.

Thus, while the composite endpoint inevitably combines objective and functional measures, each component reflects underlying neural injury. We believe our stringent adjudication protocol minimizes bias related to follow-up modality. That said, we recognize that subtle or late-emerging deficits may be underascertained, which may affect generalizability — particularly in settings with less structured follow-up. Hence, external validation with standardized neurodevelopmental assessments remains essential.

Once again, thank you for your rigorous and constructive engagement with our work. Your feedback is invaluable as we strive to develop a reliable, equitable, and clinically actionable predictive tool for this vulnerable population.

## Funding

This study was supported by the Beijing Natural Science Foundation (nos. 7,244289 and 7,232009), Beijing Municipal Administration of Hospitals Incubation Program (No. PX2024047), 10.13039/501100001809National Natural Science Foundation of China (no. 62201569), and High-level Public Health Technical Personnel Construction Project of the Beijing Municipal Health Commission (Grant no. Academic leader: −03-02).

## Data availability

N/A.

## Editor

Chandani Bhatnagar

## Declaration of competing interest

The authors declare that they have no known competing financial interests or personal relationships that could have appeared to influence the work reported in this paper.
